# Correlations between empathy fatigue, occupational identity, and sleep quality in nursing staff: a cross-sectional study

**DOI:** 10.3389/fpubh.2024.1401044

**Published:** 2024-06-12

**Authors:** Yingying Zhao, Xin Wang, Tao Liu

**Affiliations:** ^1^Department of Nursing, Jinzhou Medical University, Jinzhou, China; ^2^Department of Gerontology, Panjin Liao-Oil Field Gem Flower Hospital, Panjin, China; ^3^Department of Nursing, Huaian Hospital of Huaian City, Huaian, China

**Keywords:** nursing staff for the aged, empathy fatigue, professional identity, sleep quality, nurses

## Abstract

**Objective:**

To investigate the status quo of empathic fatigue, professional identity, and sleep quality of nursing staff in nursing institutions. To analyze the correlation between empathic fatigue, professional identity and sleep quality of nursing staff.

**Methods:**

This is a cross-sectional study. The method of convenient sampling was used to select 224 nursing workers from the older adult's institutions in the Panjin area as the investigation objects. The nurses' general data questionnaire, the Chinese version of the compassion fatigue short scale, the nurses' professional identity Scale, and the Pittsburgh Sleep Quality Index were used as evaluation tools. SPSS26.0 statistical software was used to sort out and analyze the data.

**Results:**

There was a positive correlation between empathic fatigue and sleep quality; there was a negative correlation between empathy fatigue and professional identity. Occupational identity and sleep quality were negatively correlated.

**Conclusion:**

There is a correlation between empathic fatigue, professional identity, and sleep quality of nursing workers. Empathy fatigue is positively correlated with sleep quality. Empathy fatigue was negatively correlated with professional identity. Occupational identity was negatively correlated with sleep quality. To provide a theoretical basis for the management of older adult's nursing staff and the formulation of corresponding management systems and policies, promote the mental health of older adult's nursing staff, improve sleep quality, and provide a theoretical basis and reference for future intervention research.

## 1 Introduction

According to the World Health Organization (WHO), A Decade of Healthy Aging: in its Baseline report, the world's older adult population will be just over 1 billion in 2021, or about 13.5% of the global population, and one in six of the world's population is expected to be 60 years of age or older by 2030 (1). The National Bureau of Statistics released the main data of the seventh national population census in May 2021. The results show that as of midnight on November 1, 2020, the older adult population aged 60 or above was 264 million, accounting for 18.70% of the total population. In the past 20 years since the aging society began in 2000, the proportion of the older adult population has increased by 8.4%. The degree of aging in China (2) has further deepened. The speed and degree of global aging have accelerated, and the pressure on pension services has also increased, and the demand for pension caregivers has increased. However, the research shows that older adult's care workers in China have more negative experiences in their work, such as heavy work tasks, low social status (3–6), weak family support, low professional recognition, and different degrees of sleep disorders. In the face of the new and high requirements of the combined medical and nursing institutions, the work form, work content, and work mechanism of the older adult's care workers are faced with great challenges and improvement measures should be put forward from the aspects of improving the structure of the talent team, improving the level of treatment and welfare, strengthening the sense of professional identity and value, and improving the vocational training system. To improve the work level and service quality of the nursing staff in the combined medical and nursing institutions.

Empathic fatigue (CF) (7) has been described as “a loss of ability to develop compassion.” It manifests as classic symptoms of burnout, including feelings of sadness, lack of concentration, anger, frustration, frustration, and other emotional exhaustion that leads to professional dysfunction and low productivity. The study (8) shows that empathic fatigue is prevalent in clinical nurses, up to 83.88%.

Professional identity (9) is the perception of your relationship to the work you do. Occupational identity, including the content, nature, personal value, and social significance of work, is the psychological basis for doing one's work well and achieving organizational goals. It is also the process of gradually developing self-awareness in the professional field. Several studies have shown that the influencing factors of occupational identity are related to individual factors and social factors. There is a negative correlation between turnover intention, social support, and occupational identity, and bad occupational identity will lead to an increase of turnover intention (10).

Sleep quality is a sleep index (11) that can be recorded by measuring tools as a comprehensive assessment index, including the sleep process and effect, to measure the objective sleep situation, and to assess people's subjective feelings. The adverse effects of shift work (12, 13) on sleep quality have been verified by many scholars. Yang (14) found in his research that most older adult's care workers have sleep disorders to varying degrees, which further aggravate the mental and psychological problems of older adult's care workers and further affect the quality of nursing.

Several domestic studies (15–19) have proved that there is a negative correlation between empathy fatigue and professional identity, and the level of professional identity of medical workers in China is low, and the degree of empathy fatigue is high. Several studies (20–24) on nurses have shown that improving their empathic fatigue can improve their sleep quality and promote their mental health. Rashid et al.'s (25) research shows that physicians have better empathy than surgeons, and surgeons have higher fatigue scores. Liang et al. (26) found that different levels of sleep problems would affect the utilization of social support. Coping with career setbacks can regulate the incidence of poor sleep quality.

To sum up, it is necessary to explore the correlation between empathic fatigue, occupational identity, and sleep quality of older adult's caregivers.

## 2 Objects and methods

### 2.1 Participants and procedures

The study subjects were aged care workers in older adult's care institutions in Pan Jin City from August 2022 to February 2023. Inclusion criteria: (1) Worked for more than 3 months in older adult's care institutions in the Panjin city area, caring for the older adult. (2) Voluntary participation and informed consent. Exclusion criteria: (1) Older adult's carers not in post during the survey period. (2) Older adult's carers with literacy and comprehension difficulties. A total of 245 questionnaires were distributed, with 224 valid questionnaires, and the effective rate was 91.4%.

This study is a cross-sectional study, including 13 items in the General Information Questionnaire for nursing staff, two dimensions in the short scale of empathic fatigue, five dimensions in the nurses' professional identity Scale, and five dimensions in the Pittsburgh Sleep Quality Index Scale. There are 25 variables in the questionnaire in this study. According to the rough sample estimation method proposed by Preacher and Kelley (13), the sample size should be 5–10 times the variables, and the sample size should be 125–250 cases. Considering the sample loss of 20% and convenient sampling error, the sample size should be 150–300 cases. A total of 224 effective samples were collected, which met the sample size requirements of the Kendall sample calculation method.

### 2.2 Research tools

#### 2.2.1 General information questionnaire for older adult's care workers

Refer to the general data survey scale used in Zhang's (27) survey, including the demographic characteristics of older adult's care workers, factors related to the job itself, and the work treatment of older adult's care workers.

#### 2.2.2 Chinese version of empathic fatigue short scale

Revised by Lou (28) in 2012, the scale includes two dimensions of secondary trauma and job burnout, among which the secondary trauma dimension contains five items (3, 5, 8, 10, 12). The dimensions of job burnout include eight items (1, 2, 4, 6, 7, 9, 11, 13). The scale has a total of 13 items, which are scored by Likert at 10 levels, ranging from “never/rarely” to “very frequent.” The total score of the scale is 13–130 points, and the degree of empathy fatigue is divided into three levels according to the items: 1–4 is low level, 4–7 is medium level, and 7–10 is high level. The higher the score, the higher the level of empathy fatigue. Cronbach's α coefficient total table: 0.90, burnout is 0.87, and secondary trauma is 0.83. In this study, Cronbach's α coefficient was 0.942.

#### 2.2.3 Occupational identity scale for nurse

Liu (29) based on the reference of foreign literature, through qualitative interviews and expert consultation, and combined with the actual situation in China, designed the scale by herself, including 30 items in five dimensions (cognitive evaluation, social support, social skills, frustration coping, and self-reflection), all of which are positive scores, and Likert 5-level scoring method is adopted. The scale from “very inconsistent” to “very consistent” is 1–5 points, with a total score of 30–150 points. The higher the score, the higher the level of professional identity and corresponding dimensions of nurses. The total score of the scale was 30–60, divided into the low group of professional identity, 61–90 was divided into the low group, 91–120 was divided into the medium group, and 121–150 was divided into the high group. The Cronbach's α coefficient of the total volume table is 0.938, and the broken half reliability is >0.88, which has good reliability. In this study, Cronbach's α coefficient was 0.806.

#### 2.2.4 Pittsburgh sleep quality index scale (PSQI)

In 1989, Bride et al. (30), an expert from the University of Pittsburgh, and his team developed this scale, which is a commonly used scale for comprehensive evaluation of sleep quality and quantity and can be used to evaluate the sleep quality of the research subjects in the past month. It mainly consists of 18 self-assessment items and five other assessment items, with scores ranging from 0 to 21 points. The five other assessment items asked the subjects' roommates or bed partners, which can help clinical diagnosis and treatment of sleep disorders and did not contribute to the score. The 18 self-rated items constituted seven dimensions, namely, sleep duration, sleep efficiency, self-rated sleep quality, sleep latency, sleep disturbance, sleeping pill use, and daytime dysfunction. Cronbach's α coefficient was 0.84. The PSQI total score of 0–5 indicates good sleep quality, 6–10 points indicates good sleep quality, 11–15 points indicates average sleep quality, and 16–21 points indicates poor sleep quality. In 1996, Liu et al. (11) tested that the Chinese version of the scale had good reliability and validity analysis. In this study, Cronbach's α coefficient was 0.898.

### 2.3 Ethical approval

Prior to conducting the research study, the purpose, content and significance of the study were explained in detail to the older adult's carers who met the inclusion criteria, and an informed consent form was signed after consent was obtained. The older adult's carers were informed and promised that the information obtained from this survey would be limited to this study and would not be used for other purposes, and that they had the right to choose to participate or withdraw from the study according to their own circumstances during the course of the study.

The study was approved by the Hospital Ethics Committee of Panjin Liao-oil Field Gem Flower Hospital (Approval No. KYL-2023-041).

### 2.4 Statistical methods

Firstly, the person in charge of each nursing home institution was contacted to explain the purpose and method of the study. After obtaining their consent and support, the researcher explained the purpose and method of the study to the research subjects in an offline way to ensure the quality of the questionnaire filling. After obtaining the consent of the research subjects, the questionnaire was issued and the questionnaire items and notes for filling in were informed with unified guidelines. The researchers answered the questions in time, and the time to complete the questionnaire was controlled within 20–30 min. After the questionnaire was completed, the researchers checked the filling status, timely eliminated unqualified questionnaires, and uniformly recovered them. SPSS26.0 software was used to input the original data to establish a database, and statistical analysis was carried out on the collated data. The specific statistical methods are as follows: descriptive statistical methods are used to describe the counting data; Single factor analysis was performed by *t*-test or ANOVA. Occupational identity and empathy fatigue were used as independent variables, and sleep quality was used as dependent variables for step-by-step regression analysis to establish the model.

## 3 Results

### 3.1 General information of older adult's care workers

A total of 245 questionnaires were issued in this study, of which 224 were valid, with an effective recovery rate of 91.4%. There were 77 males (34.4%) and 147 females (65.6%). 58.9% (132/224) of the participants were over 50 years old. 68.3% (153/224) of the participants had worked for 1–5 years; 59.4% (133/224) of the participants had a high school education, and 94.2% (211/224) were married. Aged care workers with advanced qualifications accounted for 58.5% (131/224); 77.7% (174/224) of the participants worked in public care institutions; 88.8% (199/224) of the participants had a monthly income of 2,000–5,000 yuan; 74.1% (166/224) of the participants worked < 8 h per day (excluding 8 h); Among them, 40.2% (90/224) and 39.7% (89/224) were satisfied or very satisfied with the older adult's care work. 63.5% (142/224) of the participants cared for 1–5 older adults persons; 34.4% (77/224) and 44.6% (100/224) of the participants' families expressed support or very support for the older adult's care work; 72.8% (163/224) of aged care workers work 1–4 night shifts per month. The general demographic data of older adult's care workers are shown in Table 1.

**Table 1:** General demographic data of older adult's care workers (*n* = 224).

**Factors**	**Group**	***N***	**%**
Gender	Male	77	34.4
	Female	147	65.6
Age (years)	< 25	12	5.4
	26–30	7	3.1
	30–35	13	5.8
	36–40	8	3.6
	41–45	31	13.8
	46–50	21	9.4
	>50	132	58.9
Years of service (years)	3 months−1	15	6.7
	1–5	153	68.3
	6–10	15	6.7
	11–15	15	6.7
	>15	26	11.6
Literacy	Elementary school	8	3.6
	Middle school	43	19.2
	Technical secondary school	11	4.9
	High school	133	59.4
	Junior college	16	7.1
	Undergrad	9	4.0
	Graduate students	4	1.8
Marital status	Unmarried	7	3.1
	Married	211	94.2
	Divorced	5	2.2
	Widowed spouse	1	0.4
Aged care worker qualification certificate levels	There is no	32	14.3
	Beginner	24	10.7
	Intermediate	33	14.7
	Advanced	131	58.5
	Technician	4	1.8
Nature of nursing institution	Public institutions	174	77.7
	Public and private	27	12.1
	Private private	23	10.3
Average monthly income	Less than 2,000 yuan (excluding 2,000 yuan)	15	6.7
	2,000–5,000 yuan	199	88.8
	5,000–8,000 yuan	10	4.5
Working hours per day	Less than 8 h (not including 8 h)	166	74.1
	8–10 h	39	17.4
	10 h and up	19	8.5
You on older adult care job satisfaction	Very dissatisfied	5	2.2
	Unsatisfied	7	3.1
	General	33	14.7
	Satisfied	90	40.2
	Very satisfied	89	39.7
The number of seniors you are caring for	1–5 older adult	142	63.4
	6–10 older adult	72	32.1
	11–15 older adult	5	2.2
	More than 15 older adult	5	2.2
Family support situation	Very unsupportive	7	3.1
	Not supportive	6	2.7
	Doesn't matter	34	15.2
	Support	77	34.4
	Very supportive	100	44.6
Number of night shifts per month	0	10	4.5
	1–4	163	72.8
	5–8	14	6.3
	>8	37	16.5

### 3.2 The status quo of empathic fatigue, professional identity, and sleep quality of older adult's care workers and the differences in general data

#### 3.2.1 Empathic fatigue scores of older adult's care workers

The total score of empathy fatigue and the scores of each dimension of the general demographic data of the study subjects were normally tested, and the data of each group followed normal distribution, the score of empathy fatigue was (37.70 ± 25.04), the score of the second dimension was (14.57 ± 9.75), and the score of job burnout was (23.13 ± 15.53). The specific results are shown in Table 2.

**Table 2:** Current situation of empathic fatigue of aged care caregivers (*n* = 224, x̄, s).

**Dimension (number of items)**	**Min**	**Max**	**x̄**	**s**
Secondary trauma (5)	5	45	14.567	9.747
Job burnout (8)	8	73	23.129	15.525
Total empathic fatigue score (13)	13	112	37.696	25.037

There were significant differences in the age and working years of the subjects, with statistical significance (*P* < 0.05). People aged 30–35 years old and working years 1–5 years old had relatively higher empathic fatigue.

#### 3.2.2 Professional identity score of nursing staff

The total score of professional identity and scores of each dimension of the general demographic data of the research subjects were tested by normality, and each group of data followed a normal distribution. The total score of professional identity was (90.43 ± 31.74), and the cognitive evaluation score was (27.21 ± 9.725) in all dimensions of professional identity. Social support (18.14 ± 6.44); Social skills (17.89 ± 6.70), frustration coping (18.20 ± 6.64), and self-reflection (8.99 ± 3.36). See Table 3 for details.

**Table 3:** Current status of professional identity of aged care workers (*n* = 224, x̄, s).

**Dimension (number of items)**	**Min**	**Max**	**x̄**	**s**
Cognitive evaluation (9)	10	43	27.210	9.725
Social support (6)	7	28	18.143	6.442
Social skills (6)	6	30	17.888	6.695
Coping with frustration (6)	7	30	18.201	6.639
Self-reflection (3)	3	15	8.987	3.361
Total professional identity score (30)	37	138	90.429	31.74

The occupational identity of the study subjects had significant differences in working years, educational level, and average monthly income, with statistical significance (*P* < 0.05). The study subjects with working years of 3 months to 1 year, postgraduate education and average monthly income of more than 5,000–8,000 yuan had relatively high occupational identity.

#### 3.2.3 Sleep quality scores of nursing staff for the aged

The total score of sleep quality and scores of each dimension of the general demographic data of the study subjects were tested by normality, and the data of each group obeyed normal distribution. Self-reported sleep quality in the present study (2.17 ± 0.73), sleep time (1.19 ± 0.65), sleep time (1.54 ± 0.59), sleep efficiency (1.496–1.1) and sleep disorders (1.68 ± 0.74), the hypnotic drug (1.13 ± 1.06), daytime dysfunction (1.44 ± 0.83), sleep quality disorder score was (10.65 ± 3.64). The specific results are shown in Table 4.

**Table 4:** Basic indexes of sleep quality of older adult's care workers (*n* = 224, x̄, s).

**Dimension (number of items)**	**Min**	**Max**	**x̄**	**s**
Self-rated sleep quality (18)	1	3	2.174	0.728
Falling asleep time	0	3	1.192	0.645
Sleep time	0	3	1.540	0.590
Sleep efficiency	0	3	1.496	1.100
Sleep disorders	0	3	1.679	0.736
Hypnotic drugs	0	3	1.134	1.063
Daytime dysfunction	0	3	1.438	0.829
Total sleep quality score	1	19	10.652	3.644

There were significant differences in the sleep quality of the study subjects in age, average monthly income, and number of night shifts per month, with statistical significance (*P* < 0.05). Subjects over 50 years old, bachelor's degree or above, average monthly income of 2,000–5,000 yuan, and an average monthly night shift number of more than 8 had a relatively high total score of sleep quality.

### 3.3 Correlation analysis of empathic fatigue, occupational identity, and sleep quality

Pearson correlation analysis was used to study the correlation between sleep quality, occupational identity, and empathic fatigue. Specific analysis showed that the correlation between sleep quality and occupational identity was −0.338, the correlation between sleep quality and empathic fatigue was 0.366, and the correlation coefficient between empathic fatigue and occupational identity was −0.492, both showing 0.01 level of significance. For details, see Table 5.

**Table 5:** Correlation between empathic fatigue, professional identity, and sleep quality of nursing care workers (*n* = 224).

	**x̄**	**s**	**Sleep disorder**	**Professional identity**	**Empathic fatigue**
Sleep disturbances	10.652	3.644	1		
Professional identity	90.429	31.74	0.338^***^	1	
Empathic fatigue	37.696	25.037	0.366^***^	0.492^***^	1

^***^p < 0.001.

With occupational identity and empathic fatigue as independent variables and sleep quality as dependent variables, the stepwise method was used for stepwise regression analysis. After automatic recognition of the model, the remaining two items of occupational identity and empathic fatigue were included in the model, and the R-square value was 0.167. The model passed the *F*-test (*F* = 22.149, *p* = 0.000 < 0.05), and the model formula was as follows: sleep quality = 11.360–0.024^*^ occupational identity + 0.038^*^ empathic fatigue. See Table 6 for details.

**Table 6:** Stepwise regression analysis of influencing factors of sleep quality of older adult's care workers (*n* = 224).

	**Non-standardized coefficient**		**Coefficient of standardization**	***t***	***p***	**VIF**	***R* ^2^**	**Adjust *R*^2^**	***F***
	***B***	**Standard error**	***Beta***						
Constant	11.36	1.007	-	11.283	0.000^***^	-	0.167	0.159	*F*_(2, 221)_ = 22.149, *p* = 0.000
Professional identity	0.024	0.008	0.208	2.948	0.004^**^	1.319			
Empathic fatigue	0.038	0.01	0.264	3.746	0.000^***^	1.319			

Dependent variable: sleep quality.

^**^p < 0.01.

^***^p < 0.001.

## 4 Discussion

### 4.1 The research object general data analysis

A total of 224 older adult's care workers were included in this study, among which female older adult's care workers (65.6%) were the main subjects. 68.3% (153/224) of the participants had worked for 1–5 years, reflecting the high turnover rate and the instability of the nursing team. 87.1% (31) (195/224) of the participants had a high school education or below, which was generally low. However, with the attention paid by the state (32) to the older adult's care work, various universities have developed senior nursing majors. This phenomenon will be gradually improved.

### 4.2 The status quo of empathic fatigue, occupational identity, and sleep quality of the subjects

#### 4.2.1 Current situation and influencing factors of empathy fatigue

The scores of empathic fatigue were (37.70 ± 25.04), the second dimension was secondary trauma (14.57 ± 9.75), and job burnout (23.13 ± 15.53). The results showed that the empathic fatigue of nursing workers was at a medium-low level. There were significant differences in age and working years (*P* < 0.05), and those aged 30–35 years and working years 1–5 years had relatively higher scores of empathic fatigue. This is related to the inconsistent management level and welfare benefits of pension institutions (33) and the imperfection of the security system. Nursing staff have long been in contact with the older adult with many basic diseases, tantrums due to illness, incontinence, and aggressive behavior, which brings a certain degree of secondary trauma to nursing staff, and the lack of necessary psychological intervention. It is different from the current situation of empathic fatigue of clinical nurses in the analysis of influencing factors (8), which is related to the good career planning and promotion mechanism of clinical nurses. However, nursing care workers have high labor intensity, low social status, insufficient number of nursing care workers, high mobility, and low professional level (34), which makes nursing care workers lack clear cognition and career planning for the industry they are in, and they are paralyzed in daily work.

#### 4.2.2 Analysis of the status quo and influencing factors of occupational identity

The total score of professional identity was (90.43 ± 31.74), and the cognitive evaluation score was (27.21 ± 9.725) in all dimensions of professional identity. Social support (18.14 ± 6.44); Social skills (17.89 ± 6.70), frustration coping (18.20 ± 6.64), and self-reflection (8.99 ± 3.36). The professional identity of older adult's care workers is at a medium level. The occupational identity of the study subjects had statistical significance in working years, educational level, and average monthly income (*P* < 0.05). The occupational identity of the research subjects who have worked for 3 months to 1 year, have a master's degree and an average monthly income of more than 5,000–8,000 yuan is relatively high. Relatively standardized pre-job training helps improve the professional identity of older adult's care workers (32). Highly educated older adult's care workers have a better understanding of the older adult's care industry and the understanding and control of national policies (35) and have clear career planning, so they have a high sense of professional identity. Nursing workers with an average monthly income of more than 5,000–8,000 yuan have a higher sense of professional identity. This is consistent with the study of Jiao et al. (36).

#### 4.2.3 Analysis of the status quo and influencing factors of sleep quality

The sleep quality score of the nursing staff was (10.65 ± 3.64). Self-reported sleep quality (2.17 ± 0.73), sleep time (1.19 ± 0.65), sleep time (1.54 ± 0.59), sleep efficiency (1.496 ± 1.1), and sleep disorders (1.68 ± 0.74), the hypnotic drug (1.13 ± 1.06), and (1.44 ± 0.83) daytime dysfunction. The overall sleep quality of the nursing staff was poor. The sleep quality of the study subjects had statistical significance in age, average monthly income, and number of night shifts per month (*P* < 0.05). The total score of sleep quality was relatively high among the subjects who were over 50 years old, had a bachelor's degree or above, had an average monthly income of 2,000–5,000 yuan, and had an average number of night shifts per month of more than 8. Older adult's care workers over the age of 50 are in the stage of physical decline, and the shift system of day and night reversal (12) leads to poor sleep quality. At present, society does not recognize highly educated people engaged in older adult's care work (37), and family support is not good, which leads to high psychological pressure on older adult's care workers with high education, and then leads to sleep disorders. With an average monthly income of 2,000–5,000 yuan, the salary level of older adult's care workers can only maintain a basic life, which is not enough to cope with emergencies in life. There is an economic crisis, and there is no clear plan for the salary increase level of older adult's care workers, resulting in anxiety among older adult's care workers, which leads to sleep disorders. Nursing staff with an average of more than eight night shifts had a higher total score of sleep quality and worse sleep quality than clinical nurses (38, 39). However, due to the serious shortage of nursing staff, the problem of excessive night shifts cannot be effectively solved for the time being. Relevant departments need to pay enough attention to improve the sleep status of older adult's care workers.

### 4.3 Correlation analysis of empathic fatigue, professional identity, and sleep quality of study subjects

Pearson correlation analysis was used to study the correlation between sleep quality, occupational identity and empathic fatigue. There was a significant negative correlation between occupational identity and sleep quality, a significant positive correlation between empathic fatigue and sleep quality, and a significant negative correlation between empathic fatigue and occupational identity.

With occupational identity and empathy fatigue as independent variables and sleep quality as dependent variables, the stepwise regression method was used to carry out stepwise regression analysis. The formula of the model is sleep quality = 11.360–0.024^*^ professional identity + 0.038^*^ empathic fatigue. The model passed the *F*-test (*F* = 22.149, *p* = 0.000 < 0.05), indicating that the model was effective. The analysis shows that empathic fatigue has a significant positive impact on sleep disorders, and occupational identity has a significant negative impact on sleep disorders. This is consistent with the conclusion reached in the study of operating room nurses conducted by Yu (40). Different from Yu Fangping's study, this study established a model and proved that occupational identity and empathic fatigue could explain 16.7% of the changes in sleep quality.

## 5 Strengths and limitations

Most of the previous studies were on the comparison of the two factors, and there were few studies on the combined analysis of the three factors. In this study, empathic fatigue, occupational identity and sleep quality were analyzed together, and the correlation analysis of the three factors was further in-depth.

In this study, the method of convenient sampling was adopted to conduct the investigation, which would reduce the external validity of the research results. In the follow-up study, the stratified sampling method can be considered to improve the representativeness of samples. This study only includes older adult's nursing staff working in older adult's care institutions in the Panjin area, and the survey results can only represent the current situation of older adult's nursing staff in the Panjin area. In addition, the cross-sectional survey conducted this time cannot infer causality due to certain limitations, and the sample size will be further expanded for future longitudinal studies.

## 6 Conclusion

There are differences in the general statistics of empathic fatigue, professional identity, and sleep quality of nursing staff. The empathic fatigue of the study subjects was at a medium to low level. People aged 30–35 years old with 1–5 years of working life had relatively high levels of empathic fatigue. The occupational identity of the subjects was at a medium level. Research subjects with working years of 3 months to 1 year, graduate education, and average monthly income of more than 5,000–8,000 yuan have a relatively high vocational identity. The subjects of this study had higher sleep quality scores but poor sleep quality. Subjects over 50 years old, with a bachelor's degree or above, an average monthly income of 2,000–5,000 yuan, and more than eight-night shifts per month had a relatively high total score of sleep quality, that is, poor sleep quality. There was a correlation between empathy fatigue, occupational identity, and sleep quality. Empathy fatigue is positively correlated with sleep quality; Empathy fatigue is negatively correlated with professional identity. Occupational identity was negatively correlated with sleep quality.

## Data availability statement

The raw data supporting the conclusions of this article will be made available by the authors, without undue reservation.

## Ethics statement

The studies involving humans were approved by the Ethics Committee of Pan Jin Liao You Baoshihua Hospital. The studies were conducted in accordance with the local legislation and institutional requirements. The participants provided their written informed consent to participate in this study. Written informed consent was obtained from the individual(s) for the publication of any potentially identifiable images or data included in this article.

## Author contributions

YZ: Writing – review & editing, Writing – original draft, Visualization, Validation, Software, Resources, Methodology, Investigation, Formal analysis, Data curation, Conceptualization. XW: Writing – review & editing, Project administration, Methodology, Investigation. TL: Writing – review & editing, Supervision, Project administration.
